# Host–Bacterium Interaction Mechanisms in *Staphylococcus aureus* Endocarditis: A Systematic Review

**DOI:** 10.3390/ijms241311068

**Published:** 2023-07-04

**Authors:** Francesco Nappi, Sanjeet Singh Avtaar Singh

**Affiliations:** 1 Department of Cardiac Surgery, Centre Cardiologique du Nord, 93200 Saint-Denis, France; 2 Department of Cardiothoracic Surgery, Royal Infirmary of Edinburgh, Edinburgh EH16 4SA, UK; sanjeet.singh@glasgow.ac.uk

**Keywords:** infective endocarditis, *Staphylococcus aureus* infection, *Staphylococcus aureus* immunity, *Staphylococcus aureus* cytotoxin, biofilm resistance, host innate immunity

## Abstract

*Staphylococci* sp. are the most commonly associated pathogens in infective endocarditis, especially within high-income nations. This along with the increasing burden of healthcare, aging populations, and the protracted infection courses, contribute to a significant challenge for healthcare systems. A systematic review was conducted using relevant search criteria from PubMed, Ovid’s version of MEDLINE, and EMBASE, and data were tabulated from randomized controlled trials (RCT), observational cohort studies, meta-analysis, and basic research articles. The review was registered with the OSF register of systematic reviews and followed the PRISMA reporting guidelines. Thirty-five studies met the inclusion criteria and were included in the final systematic review. The role of *Staphylococcus aureus* and its interaction with the protective shield and host protection functions was identified and highlighted in several studies. The interaction between infective endocarditis pathogens, vascular endothelium, and blood constituents was also explored, giving rise to the potential use of antiplatelets as preventative and/or curative agents. Several factors allow *Staphylococcus aureus* infections to proliferate within the host with numerous promoting and perpetuating agents. The complex interaction with the hosts’ innate immunity also potentiates its virulence. The goal of this study is to attain a better understanding on the molecular pathways involved in infective endocarditis supported by *S. aureus* and whether therapeutic avenues for the prevention and treatment of IE can be obtained. The use of antibiotic-treated allogeneic tissues have marked antibacterial action, thereby becoming the ideal substitute in native and prosthetic valvular infections. However, the development of effective vaccines against *S. aureus* still requires in-depth studies.

## 1. Introduction

In many high-income countries, virulent staphylococci represent the leading causative pathogens of infective endocarditis (IE) overtaking penicillin-sensitive streptococci. [1,2,3]. Similarly, the subjects at risk of contracting IE by staphylococcus bacteremia have increased the burden on healthcare facilities, and tackling this infection represents one of the paramount challenges of infection in the 21st century [4,5,6]. This concern is related to the biomolecular characteristics of a *Staphylococcus aureus* infection, which often has increased resistance to many antibiotics, constituting a major conundrum in modern healthcare [7,8,9]. *Staphylococcus aureus* interacts with the host’s innate immunity, playing a pivotal role in sustaining and maintaining the infectious state. The pathogen generates a protective shield that interferes with the host’s protective mechanisms using two coagulases, the von Willebrand factor binding protein (vWFbp) and Coagulase (Coa), leading to its virulence [10,11,12,13,14,15]. These molecules make up a functionally intricate framework that offers *S. aureus* a defensive shield as a result of the assembly of a fibrinogen/fibrin complex to surround the pathogen and generate large vegetations. A substantial concern for staphylococcal infections is related to the specific characteristics of these vegetations. These can be large, mobile, and very frequently located in the mitral valve; this phenomenon has been linked to a markedly increased risk of symptomatic embolic events [16,17,18,19]. Although in 50% of patients, embolic events occur subtly and asymptomatically, in up to 80% of patients, magnetic resonance imaging (MRI) of the brain may highlight cerebral injuries [20,21,22]. The former condition may generate mycotic aneurysmal lesions resulting from a septic arterial embolism, which is associated with the migration of the pathogen to the intraluminal space or vasa vasorum, followed by the diffusion of the infection through the vascular structure. Mycotic aneurysms were recorded in 5% of IE, especially in older patients with weakened immunity to *S. aureus* infections. Recently the detection of lesions is more frequently recorded through the increasing use of advanced imaging methods [23,24,25,26]. To date, the literature lacks systematic reviews that have evaluated the host–bacterium interaction mechanisms in *Staphylococcus aureus* endocarditis. This systematic review aims to offer a broader understanding of these interaction mechanisms. The immune response process, the interactions with coagulation mechanisms, and biofilm formation were investigated. We believe that the data presented here could provide a basis for the further consistent evaluation of *Staphylococcus aureus* endocarditis and assist the doctor–patient discussion on the benefits and expectations of potential new therapeutic approaches.

The infection sustained by *Staphylococcus aureus* concerns the clinical epidemiological context as well as a pathophysiological one. An ongoing effort is being made by scientists to better understand the molecular mechanisms responsible for infection and the corresponding immune response. Considering the clinical–epidemiological domain (upper panel), infective endocarditis in high-income countries recognizes *Staphylococcus aureus* as the main causative pathogen. Coagulase-negative staphylococci form the infectious fields in only 9.7% of recorded infections compared to 26.6% overall. The population of individuals most affected by the infection is over 65 years of age and are often recipients of a CIED implant. Blood cultures should be routinely performed before starting antibiotic therapy, especially in infections caused by strains of methicillin-resistant *Staphylococcus aureus* (MRSA), which are associated with increased virulence. The mechanism of infection of eukaryotic cells induced by *S. aureus* involves the sharing of extracellular adherence protein (EAP), fibronectin-binding proteins (FnBPS), and plasmin sensitive protein (Pls), which are expressed in MRSA (lower panel). *Staphylococcus aureus* infection is promoted by the action of fibrinogen and the membrane structural protein integrin (α5) β1.

## 2. Methods

### 2.1. Search Strategy

In January 2023, the systematic review was designed using PubMed, Ovid’s version of MEDLINE, and EMBASE, and the databases were investigated using the terms “*Infective Endocarditis* coupled to *Staphylococcus aureus* Infection (9.716 to the present)”, “*Staphylococcus aureus* immunity (1.102 to the present)”, “*Staphylococcus aureus* cytotoxin (300 to the present)”, “*Staphylococcus aureus* coagulation (455 to the present)”, and “*Staphylococcus aureus* biofilm (884 to the present)”. The search was directed to prioritize the identification of data from randomized controlled trials (RCT), meta-analysis, observational cohort studies, and basic research articles. The review was registered with the OSF register of systematic reviews and followed the Preferred Reporting Items for Systematic Reviews and Meta-analyses (PRISMA) reporting guidelines. A DOI is available for the project online (https://osf.io/mnu9s, accessed on 1 January 2023).

### 2.2. Study Selection and Data Extraction

Relevant abstracts were searched (11,573), and after deduplication, 4722 relevant citations were screened. The predefined inclusion criteria guided the review of titles and abstracts. Articles in English based on infective endocarditis, *S. aureus* infection, mechanisms related to *S. aureus* immunity, and mechanisms of action of *S. aureus* cytotoxin were included. Furthermore, particular attention was given to the pathophysiology of the biofilm formation and to the mechanisms of resistance to *S. aureus* infection. Pertinent animal model studies were selected, as the topics raised improved understanding of the role played by *S. aureus* as a causal pathogen in promoting infection and shed light on the interaction between *S. aureus*, the immune response, and the coagulation process. Case reports, conference presentations, editorials, and expert opinions were excluded.

### 2.3. Endpoints and Effect Summary

The endpoints assessed the effects of the prominent role of *Staphylococcus aureus* immunity, conferring particular attention to hosts’ innate immunity, immune modulation, B-cell vs. T-cell cooperation, and immune response and vaccine. We also investigated new evidence from the infectious array of *Staphylococcus aureus* focusing on the involvement of the protective shield and host protection functions, the interaction between infective endocarditis pathogens, vascular endothelium, and blood constituents with particular attention to the role of the biofilm.

## 3. Results and Discussion

**Figure 1:**
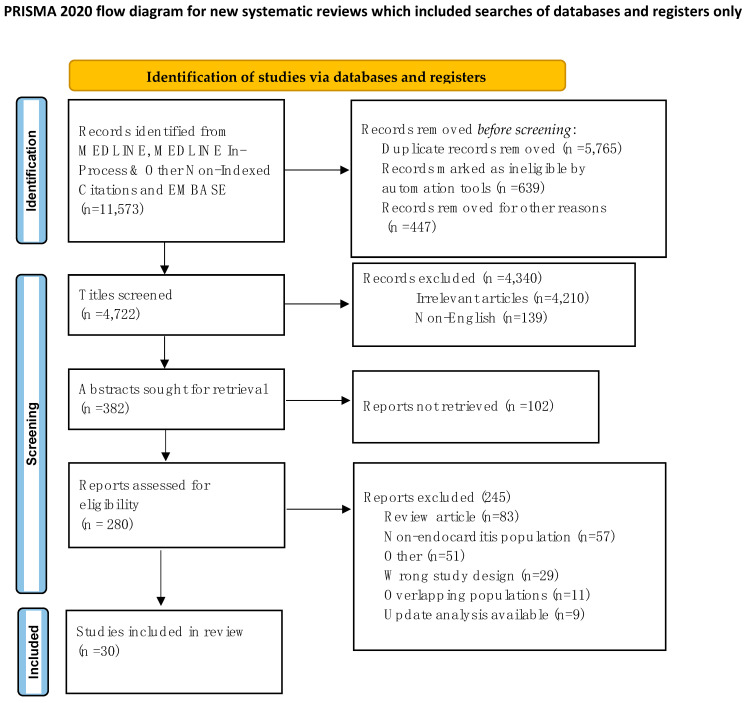
PRISMA 2021 Flow diagram for new systematic review, which include searches of databases and registers only.

A total of 280 studies were evaluated, of which 40 studies were included and 240 were excluded in the final analysis as not meeting eligibility criteria. The full PRISMA flow diagram outlining the study screening process is reported in Figure 1. The PRISMA 2020 Checklist is enclosed in the reporting checklist in the Supplementary Material. The details of the eligibility criteria for manuscripts are reported in Table 1, Table 2 and Table 3. The study design that was performed aimed to avoid heterogeneity of causes, but a trend towards higher outcome estimates was noted in small studies. The duration of the follow-up did not affect the study results. The risk of bias arising from missing results was addressed by the direct exclusion of studies that did not report results and measures of interest. We only selected studies with authors including an expert in statistics and set confidence levels at 95% and *p*-value thresholds at 0.0.

### 3.1. Staphylococcal Manipulation of Host Immune Responses

Gram-positive cocci of the staphylococcus, streptococcus, and enterococcus species are accountable for 80–90% of infective endocarditis. *S. aureus* is the commonly isolated pathogen of IE in high-income countries, accounting for up to 30% of infection events [1,2,3,4,5,6,27,28,29]. The lineage of coagulase-negative staphylococci, including *Staphylococcus epidermidis*, *Staphylococcus lugdunensis,* and *Staphylococcus capitis,* stands out as far-reaching skin commensals. Coagulase-negative staphylococci maintain distinct characteristics involving the frequent colonization of indwelling lines and CIEDs. Moreover, they are highly recurrent and are the common causative bacteria in patients with early prosthetic valvular endocarditis [30,31,32,33,34]. These pathogens are often the cause of hospital-acquired native valvular endocarditis. [35,36,37] Furthermore, coagulase-negative staphylococci may generate biofilms that can cause high rates of abscess formation and multi-antibiotic resistance [36]. IE caused by staphylococcal outbreaks affects a particular population of patients given the specificity of the immune response to the infection and the capacity to develop resistance to antibiotics. These include at-risk hemodialysis patients and intravenous drug users, but also those with native valves, prostheses, and cardiac implantable electronic devices (CIEDs) [38,39,40,41,42,43]. *Staphylococcus* sp. has an ingrained tendency to increase antibiotic resistance with methicillin-resistant strains emerging as a serious concern worldwide [2,44,45].

**Figure 2:**
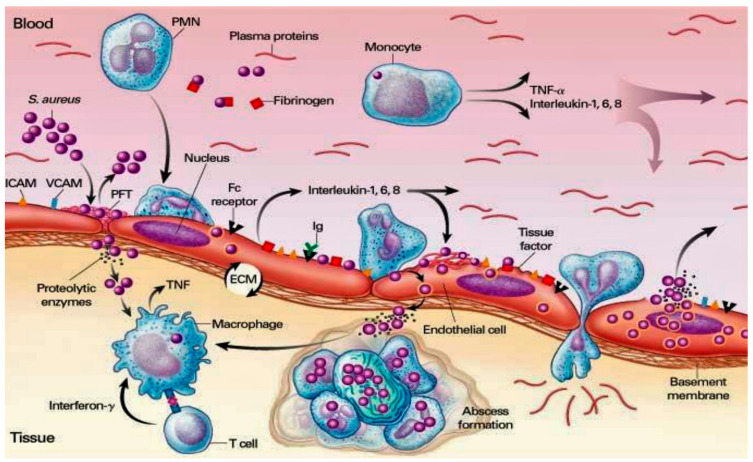
Depicts the mechanism of bacterial adhesion. The first pathophysiological process leading to IE is the development of proinflammatory cell lines such as PMN, monocyte, and macrophage is supported by the production of cytokines (TNF, α, interleukine 1, 6, and 8), adhesion molecules (ICAM, VCAM), integrins, and tissue factor. These mediators of inflammation draw monocytes and platelets through the intervention of chemokines with the associated production of fibronectin. *S. aureus* releases cytotoxins that trigger the immune response both innate and mediate (T-cell and B-cell). Abbreviations: IE, infective endocarditis; ICAM, Inter Cellular Adhesion Molecule; *S. aureus*, Staphylococcus aureus; TNF, tumor necrosis factor; VCAM, vascular cell adhesion molecule. License No 5443730915162 NEJM htttps://doi.org/10.1056/NEJMra1216063 RLNK504888203.

In the immune response advocated by pathogens without heart disease, the cardiac endothelium is not subject to recurrent bacteremia. However, the latter can be promoted by ordinary quotidian activities, routinely depicted by brushing teeth and chewing [46]. Bacterial fastening to the tissue appoints one of the crucial steps in the pathophysiological process of IE. Once the endothelial injury is initiated, bacterial adhesion is promoted through two main steps. Initially, the release of inflammatory cytokines associated with tissue factors is recorded. Following this, expression of fibronectin is observed, which advocates the generation of a thrombus constituted by a conglomerate of fibrin and platelets [47,48,49]. Common causative pathogens implicated in the development of ED can colonize heart valves either with pre-existing sterile vegetations or in the presence of the slightest endothelial injuries. The superimposed inflammatory response induces the assembly of cytokines, integrins, and tissue factors, which in turn attract monocytes and platelets. Due to the effect induced by chemokines, the combined production of fibronectin can be observed. The crucial action of the chemokines allows the bacteria to adhere, further favoring the activation of the inflammatory cascade, which offers, through the incorporation of the bacteria, an anomalous protection mechanism by the host defenses [48,49] (Figure 2).

The pathoanatomy of IE is characterized by three factors that are addressed towards the endothelium: the direct activity of the bacterial pathogen, valvular sclerosis, and/or rheumatic valvulitis. The former is strongly advocated through the interaction of *S. aureus* at the site of infection [50]. The pathophysiological and clinical assessments of IE involving heterogeneous cohorts of subjects range from individuals treated successfully without experiencing adverse events to subjects who instead showed serious complications with raised mortality rates. As there has been a modification in the temporal trend in the pattern of infective endocarditis in developed countries over the past five decades, the study of pathophysiology and clinics has involved increasingly aging subjects. These contract IE with increasing incidence of *Staphylococcus aureus* as the causative bacterium and often the infection develops within the health care setting. From this, physicians have acquired a greater understanding of the mechanisms that support the formation, growth, and embolization of vegetation that occur on damaged or inflamed heart valves to cardiac devices. Improved knowledge of these mechanisms has led to a greater understanding of how to address the growing problem of antimicrobial resistance.

Two mechanisms causing IE have been shown to play a substantial role in its treatment: the modulation of the immune response in older patients with IE and the use of new platforms for the treatment of structural pathologies of the heart such as the transcatheter procedure for valve replacement or repair that can trigger septic shock. The latter can lead to a substantially increased risk of death in patients with IE [51,52,53,54,55].

### 3.2. Subversion of Innate Immune Responses

The peculiar virulence of *S. aureus* is due to the presence of specific factors, present both on the surface of the bacterium and in its secretory molecules. Both, once triggered, give the bacterium a greater ability to counteract the host’s immunity [56,57]. *S. aureus* has a crucial virulence program, the Accessory Gene Regulatory System (AGR), which operates for the quorum detection of pathogens. Our knowledge suggests that AGR manipulates the control of the expression of phenol-soluble modulins (PSM), which are effective against immune cells and keratinocytes (KC). However, how and when this mechanism is triggered has not been fully understood [58]. The innate immune response supports a reaction by dead KCs, which generates a physical fence exerted by the deliverance of antimicrobial molecules, such as cathelicidins, human β-defensins 2 and 3, and RNase 7 while bacteriostasis against *S. aureus* infection is promoted.

Two independent studies [59,60] reported the antibacterial role of KCs that are also mediated by pattern recognition receptors (PRRs), such as toll-like receptors (TLRs) and nucleotide-binding oligomerization domain (NOD) proteins. Molecular patterns associated with invading pathogens (PAMPs) are integrated into these two surveillance systems, thus encouraging a timely defense against *S. aureus* [59,60]. In addition, the innate immune response is sustained by the activity of other cells, such as B and T cells, plasma cells, natural killer (NK) cells, dendritic cells, macrophages, mast cells, and fibroblasts individualized in the dermis [61,62]. *S. aureus* infections are promoted by several processes by which the breach of innate immune system triggers are instituted. Two other phases have also been observed from when the pathogen enters the bloodstream and subsequently spreads into the host tissue once it leaves the bloodstream. Both stages are strictly connected to the activity of specific molecules expressed by *S. aureus*, which work alongside the endothelium, the blood, and the extracellular matrix. With a well-defined role, FnBPA and FnBPB bind fibronectin and work alongside α5β1 integrin on the surface of the vascular endothelium, causing transmigration and cell invasion. Subsequently, along with wall–wall teichoic acid (WTA) and lipoteichoic acid (LTA), which are expressed as polymers in the outer envelope of *S. aureus*, the invasion of host cells is promoted. The second step of *S. aureus* infections is facilitated by the production of fibrin thrombi across the trigger of the agglutination mechanism induced by Coa/vWbp and ClfA. Binding to a von Willebrand factor (vWF) on endothelial surfaces leads to the formation of polymers, such as Ultra Large vWF (ULVWF). The third phase of *S. aureus* infections is typically characterized by the secretion of Hla, a toxin that works alongside the ADAM10 receptor, which leads to a disruption of the physiological barrier function exerted by the vascular endothelium. Lastly, due to the activation of a Trojan horse model, neutrophils containing intracellularly engulfed *S. aureus* lose the ability to deliver bacteria into host tissues [10,11,12,13,14,63].

Since *S. aureus* is devoted to interacting with immune cells during infection, the pathogen’s delivery of cytotoxins is decisive and includes leukocidins, hemolysins, and PSM. The leukocidin family comprises leukotoxins such as gamma hemolysin with HlgAB, HlgCB, LukED, and LukAB as well as Panton-Valentine Leukocidin (PVL). Three independent studies have clearly described the role played by leukotoxins [64,65,66]. Malachova et al. [64] suggested that LukAB was worthwhile only on human polymorphonuclear leukocytes (PMNs) and can destroy monocytes, macrophages, and dendritic cells. This evidence was corroborated by Alonzo et al. [65,66], who demonstrated that LukED recognizes C-C chemokine receptor 5 expressed on the cell resulting in the elimination of lymphocytes, macrophages, and dendritic cells.

At the micromolar level, the intervention of PSM and alpha-hemolysin (Hla) operates with a considerable capacity to destroy neutrophils after phagocytosis [67]. Thus, it can modulate the action of disintegrin A and metalloprotease 1 (ADAM1) and promote the eradication of monocytes, macrophages, neutrophils, and T cells [68]. A substantial role is offered by cytotoxins that serve functionally as a Trojan horse to encourage the diffusion of *S. aureus*. Foster and colleagues [69] observed that this activity is separate from the role offered by *S. aureus* in evading the host’s immune response. Cytotoxins function by notably dampening both the innate and adaptive immune responses, protecting *S. aureus* through its movement in the host.

The pathophysiology by which *S. aureus* circumvents the host immune surveillance is umpired by the protein suppressor of phytochrome A-105 (SpA proteins), which are embedded in the wall structure of *S. aureus*. These molecules form during the growth of the bacterium. The existence of five domains in the SpA, which are implied with the linkage of immunoglobulins, was demonstrated. Silverman and colleagues [70] observed that the five immunoglobulin-binding domains tie to the IgG Fcγ domain and the Fab domain of the VH3 IgG as well as to the IgM clan. This function is guided by the cross-links of the B cell receptors, which promote the polyclonal proliferation of the B cells, thus advocating the undertaking of the superantigen SpA. It primarily carries out this function during the different phases of the infection with a differing response noticed, resulting in varying expression of SpA. This event contributes to the delivery of the Hla toxin, which triggers the activity of specific B lymphocytes detected in sites far from the *S. aureus*. The described phenomenon is the immunological elucidation for which humans mostly generate antibodies resistant to Hla despite most of the detected SpA strains. Another important point to consider is linked to the fact that the Hla deliverance function is also umpired from the cell wall of the bacterium [70]. The superantigenic activity exercised by SpA proteins can be a target for future vaccines. A specific effect of SpA proteins that evade recognition by B cells has been suggested by promoting a state termed “lethargy”—a usual early response to the antigen. In this case, the B lymphocytes may not pick up a secondary signal to sustain their activation advocating a state of shock termed “anergy”. The latter is a phenomenon that arises in the colonization of *S. aureus*, in the perseverance of its infective momentum, and in the weakening of the defensive protection of T-lymphocytes caused by an impairment of their recruitment by superantigens and cytotoxins, which leads to a reduced affinity for antibodies [71,72].

### 3.3. Host–Bacterium Interaction Mechanisms in Staphylococcus aureus Endocarditis

One avenue of the pathogenic role of *S. aureus*, which is mediated by adhesion proteins, such as the fibronectin-binding protein and staphylococcal aggregation factors A and B, should be recognized. These molecules deploy the role of bacterial mediators of adhesion and are determinants for bacterial pathogenicity [73,74,75,76,77]. Likewise, the contribution offered by the induced experimental endocarditis in the animal models was of higher importance in demonstrating the pathological role sustained by the expression of *Staphylococcus* adhesins in *Lactococcus lactis*. Clumping factor A (ClfA) and fibronectin-binding protein A (FnBPA) have been suggested to play a crucial role in valve colonization [73].

Que and colleagues [78] studied the development of infective endocarditis in an animal model over three days. Successful colonization of damaged valves by ClfA-positive lactococci was observed. Removal of the infection was noted spontaneously within 48 h. FnBPA-positive lactococci showed titers of pathogens that were progressively enhanced in both the vegetation and spleens. The imaging results disclosed that whilst the ClfA-positive lactococci were confined to the vegetation, the FnBPA-positive lactococci had spread to the contiguous endothelium. This explained the ability of FnBPA to trigger cell internalization in vitro. FnBPA conveys either fibrinogen and fibronectin binding domains, so the activity of these two selective functionalities in advocating infection was evaluated by dispossessing FnBPA of the fibrinogen binding domain and incorporating it with the fibrinogen binding domain of ClfA in cis or trans configurations. Although the withdrawal of the fibrinogen binding domain of FnBPA did not modify fibronectin binding and cellular internalization in vitro, it strongly determined the dismissal of valve infectivity in vivo. Interestingly, the propensity for causing infections was resumed in the cis configuration by inserting the fibrinogen binding domain of ClfA into truncated FnBPA whilst in trans, it was reached by co-expressing full-length ClfA and truncated FnBPA by using two distinct plasmids. It may be argued that in *S. aureus* infections the binding of fibrinogen and fibronectin might contribute to valve colonization and endothelial encroachment in vivo [73].

A *Staphylococcus aureus* infection is supported by bacteremia, which not only drives complications, such as infective endocarditis and osteomyelitis, but promotes the exit of the pathogen from the bloodstream to cause metastatic abscesses. The bacterial interaction with endothelial cells works a considerable role in promoting these complications. At this stage of the infection, several bacterial proteins are implied. A fundamental role is provided by the extracellular adhesion protein (Eap) of *S. aureus*, which has many functions, including that of binding numerous host glycoproteins [77,78,79,80,81].

The Eap complex of *S. aureus* has also been observed to exert both pro- and anti-inflammatory activity. Issues have emerged in robustly evaluating the role of Eap in vivo due to the difficulties shown in defining its assets in mutant strains. There is evidence of the pro-inflammatory role of Eap and the activity that purified native adhesion protein of *S. aureus* has in triggering the delivery of TNFα in human whole blood in a dose-dependent mode. TNFα generation advocated *S. aureus* adhesion to endothelial cells with a 4-fold increase through a mechanism requiring protein A on the bacterial surface and gC1qR/p33 on the surface of endothelial cells. This finding suggested that Eap’s contribution to disease during the course of *S. aureus* bacteremia is decisive. It was genetically engineered for an isogenic set of strains, in which the Eap gene was inactivated and integrated after inserting an intact copy of the gene elsewhere on the bacterial chromosome. Using a mouse bacteremia model, Eap-expressing strains had a more serious infection, advocating the pivotal role of Eap in invasive disease [78,80,81].

**Figure 3:**
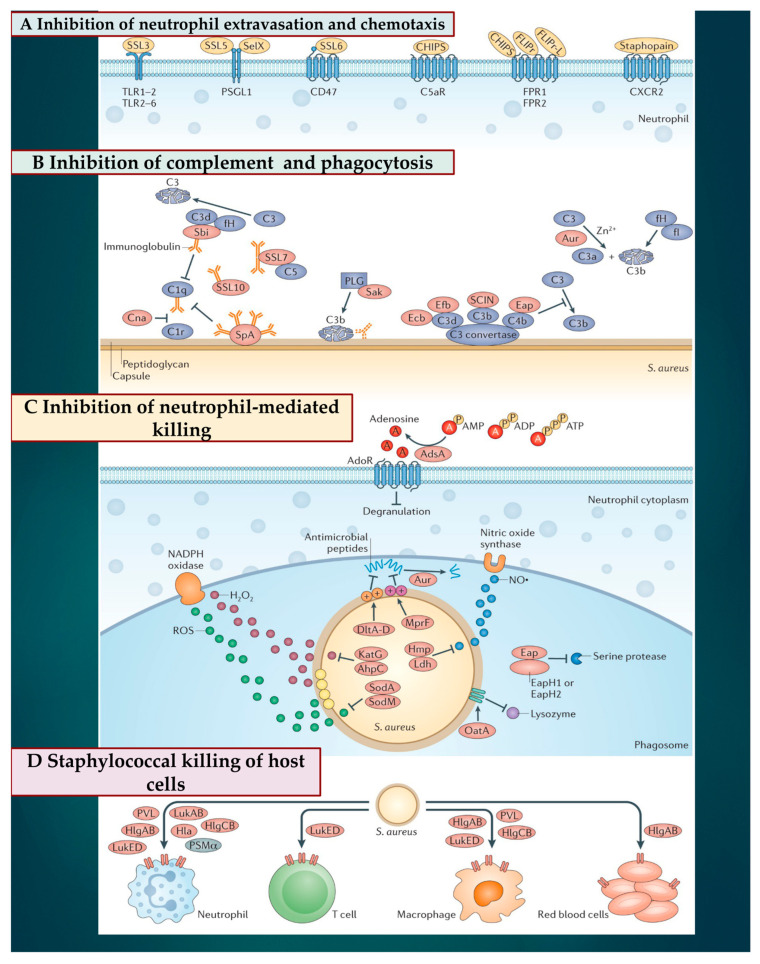
Four steps of the pathophysiology of *S. aureus* in interfering with chemotaxis, complement, and killing by phagocytes are depicted. Invoice number Invoice NRLNK 5573760477874. (**A**) *S. aureus* promotes the inhibition of neutrophil extravasation and chemotaxis by means of the secretion of staphylococcal superantigen-like (SSL) molecules. SSL3 leads to inhibition of Toll-like receptor (TLR) heterodimers, SSL5, SSL1. In addition, SelX hampers PSGL1 signaling and SSL6 impedes the interaction between G protein-coupled receptor CD47. Among the other active secreted proteins, we recognize the *S. aureus* chemotaxis inhibitory protein (CHIPS), which hinders the interaction with the complement receptor C5aR. We still find Formyl peptide 1 (FPR1) and FPR2 receptor, Formyl peptide receptor-like 1 inhibitor (FLIPr), and FLIPr-like (FLIPrL), which hinder the action of FPR1 and FPR2. Staphopain instead works by inhibiting signaling from the C-X-C chemokine receptor 2 (CXCR2). (**B**) The interference with opsonization is mediated by the secretion of inhibitory factors, which interfere with the activation of the complement factors C1q and C1r, compromising the phagocytosis of staphylococci. Specifically, collagen adhesin (Cna) blocks the association of the immunoglobulin-bound complement factor C1q with the complement receptor C1r. Staphylococcal protein A (SpA) and staphylococcal immunoglobulin ligand (Sbi) that bind to the immunoglobulin block its association with C1q. Sbi, SpA, SSL7, and SSL10 sequester immunoglobulins to block their ability to promote complement activation. Sbi (when associated with host factors C3d and factor H (fH)) and SSL7 also inactivate complement factors C3 and C5, respectively. Sak associates with plasminogen (PLG) and activates zymogen to cleave complement factor C3b and immunoglobulin. Extracellular complement binding protein (Ecb), extracellular fibrinogen binding protein (Efb), staphylococcal complement inhibitor (SCIN), and extracellular adherence protein (Eap) inhibit C3 convertases and aureolysin (Aur) cleave complement factor C3, which impairs opsonization because the C3b cleavage product is degraded by a complex of host proteins fI and fH. (**C**) *S. aureus* prevents the neutrophil-mediated killing of phagocytosed bacteria through the expression of several enzymes and inhibitors. The adenosine synthesis enzyme AdsA helps block granulation via adenosine receptor (AdoR) signaling. Staphyloxanthin, superoxide dismutase A (SodA) and SodM, catalase KatG, and alkyl hydroperoxide reductase (AhpC) are antioxidants that induce oxidative stress promoted by phagosomal reactive oxygen species (ROS) and H2O2 generation. Aureolysin (Aur) cleaves antimicrobial peptides and DltA-DltD leads to d-alanyl esterification of teichoic acids to protect staphylococci from antimicrobial peptides. MprF alters phosphatidylglycerol with alanine or lysine, another mechanism to protect staphylococci from antimicrobial peptides. l-Lactate dehydrogenase (Ldh) and flavohemoglobin (Hmp) inhibit nitrosative stress; Eap and its homologues EapH1 and EapH2 inhibit neutrophil serine proteases and OatA O-acetylated peptidoglycan, which prevents its lysozymal degradation. (**D**) Secreted β-barrel pore-forming toxins (β-PFTs) bind specific receptors on immune cells to impair immune cell functions or advocate cell lysis. These β-PFTs include leukocidin ED (LukED) that ties to neutrophils, T cells, and macrophages; γ-haemolysin AB (HlgAB) that ties to neutrophils, macrophages, and red blood cells; HlgCB and Panton–Valentine leukocidin (PVL) that attach to neutrophils and macrophages; and LukAB and α-haemolysin (Hla) that adheres to neutrophils. Phenol-soluble modulin-α (PSMα), which is another factor secreted by *S. aureus* but not a β-PFT, can also lyse leukocytes.

Bacterial colonization provides the trigger for additional cycles of endothelial harm and thrombus deposition resulting in the implantation of infected vegetations. In this stage, the formation of a biofilm, which is generated by a multilayer bacterial aggregate containing a polysaccharide combined with a protein matrix, assists bacterial persistence and contributes to antibiotic tolerance [82]. In Figure 3, staphylococcal manipulation of host immune responses is disclosed.

### 3.4. Immuno-Response and Vaccine

The spread of an antagonistic vaccine towards *S. aureus* is a crucial challenge that would allow the emergence of antibiotic-resistant strains to be addressed. Resistance to antibiotic therapy has made it possible to direct research toward alternative treatments, such as the use of immunotherapeutic drugs. However, better knowledge of the mechanisms driving the immune response during *S. aureus* infection and the manufacturing of an active vaccine are two parallel paths. In several published reports, based on infected mouse models, the ability of the *S. aureus* vaccine antigen has been evaluated to elicit an immune response that can be scaled up to safeguard multiple mouse models infected with various strains of the bacterium. This procedure allowed scientists to evaluate cross-immune protection across diverse models and with the appearance of unlinked strains of *S. aureus* [83,84,85,86].

Considerations related to the progress achieved by a successful immuno-humoral response may be mitigated by converging immune-evasion mechanisms of *S. aureus*. Given the experiences accumulated to date regarding the immune response to staphylococcal infections, there is no doubt that the progress needed to obtain a promising vaccine in terms of effectiveness and safety to *S. aureus* apparatus relies on an even better understanding of the immunity, both innate and adaptive. We learned that the immune response to *S. aureus* is articulated on the effectiveness of the humoral response, T cell function, stopping complement proteins function, and attenuating immune mediators by its toxins. The main contrasting mechanism exerted by *S. aureus* to the host concerns the ability of the pathogen to hinder the immune action. Precisely, this peculiar characteristic epitomizes the main factor responsible for the lack of success in the progress of targeted vaccines. Thus, the core problem can be related to the evolution of immunological interventions that are capable of fruitfully hampering the mechanisms by which *S. aureus* restrains immunity. This procedure could guarantee promising outcomes in vaccine spread [83,84,85,86].

A line of investigative speculation has been the role of ESAT-6-like proteins secreted by *S. aureus,* designed as *S. aureus* EsxA (SaEsxA) and SaEsxB, which have been studied as possible targets for vaccines. Although tall titers of anti-SaEsxA and anti-SaEsxB antibodies were generated in mouse models vaccinated with the administration of purified proteins (a finding revealing an antibody-mediated immune response), the *S. aureus* infection was not prevented. However, mice processed with the usage of recombinant SaEsxA (rSaEsxA) and rSaEsxB recorded sustained immunity to Th1 and Th17. Additionally, this cohort was observed to have considerably improved survival rates when subjected to *S. aureus* with respect to the control cohort. This evidence elucidated the functioning of SaEsxA and SaEsxB as two hopeful Th1 and Th17 antigen candidates, with the likelihood of future expansion towards the development of multivalent and serotype-independent vaccines hostile to *S. aureus*-induced bacteremia [84].

Brady et al. [85] focused on the genetically inactivated mutant HlaH35L of toxin alpha and analyzed the protection provided by these antigenic molecules in three infection models using the same vaccine quantity, regimen, immunization route, challenge strain, and adjuvant options. The use of a systemic infection model challenged by HlaH35L immunized mice revealed a small but statistically remarkable reduction in bacterial colonization juxtaposed to that noted in control mice. In contrast, using a prosthetic implant model of chronic biofilm infection, no notable discrepancies in bacterial standards compared to checks were observed. These results suggest that although vaccines may protect from one form of *S. aureus* disease, they seem to be inactive in providing an effective defense versus various manifestations of the disease, thus underscoring the significant challenge that exists in vaccine development against *S. aureus* [85].

Epidemiological studies have revealed the high colonization potential that characterizes *S. aureus*, between 20 to 80% in humans. This implies the potential to generate a variety of diseases that constitute a nightmare for healthcare-associated and community-associated bacterial infections [83,86]. It is evident that in such a context the development of the vaccine against *S. aureus* has been burdened by abortion, producing failures every time its enforcement has been endeavored to date. However, the reason for this failure may be due to incomplete knowledge of the tools that support the immune defense resistant to this bacterium. In humans, *S. aureus* advocates bacteremia with the potential to progress to sepsis. The genesis of infectious fields can promote endocarditis, osteomyelitis, pneumonia, and meningitis, as well as skin and soft tissue infections. People who are vectors of *S. aureus* are at an increased risk of infection and conveyance of bacteria to others. The diffusion of multidrug-resistant strains of *S. aureus* restricts first-line medical treatment through the administration of effective antibiotics [83,86].

Zhang et al. suggested a multipronged B cell-, Th1-, and Th17-mediated response averse to *S. aureus* antigens. Similarly, this precise immune response provides increased and extensive protection versus *S. aureus* by anticipating the stage of invasive infection, mucosal colonization as well as skin and soft tissue infection [86]. Today, the impact of immunotherapy is continuously cultivated and sustained and can also be indefinitely conferred by the administration of the vaccine hostile to *S. aureus* bacteremia. A decisive part is offered by *S. aureus* manganese transport protein C (MntC). This protein is a highly conserved cell surface molecule that may evoke safeguarding immunity versus *S. aureus* and *Staphylococcus epidermidis.* Wei et al. evaluated the humoral immune response and CD4+ T cell-mediated immune responses, disclosing a vital defense for mice to decrease the incursion of *S. aureus* that was supported by MntC-specific antibodies. The findings firmly underpinned the definite role of MntC-induced immunity response, disclosing that Th17 works substantially in counteracting *S. aureus* infections. Again, the evidence noted that MntC-specific antibodies and MntC-specific Th17 cells work side-by-side in forestalling *S. aureus* infections. Rather, Yu and colleagues [87] observed that MntC-promoted protective immunity declined following the neutralization of IL-17 by the antibody in vivo. Thus, adoptive Th17 from mice may not be fully refractory to the *S. aureus* challenge (Table 1).

**Table 1:** Characteristics of the Included Studies.

First Author/Year/Ref	Type of Study	Cohort	Aims	Finding
Lockhart et al. (2008) Circulation [46]	Human RCT Single Center (USA)	290 pts Brushing Gro 98 vs. Extraction-Amoxicillin 96 vs. Extraction-Placebo 96	To compare the incidence, duration, nature, and magnitude of IE-related bacteremia from single-tooth extraction and toothbrushing. To determine the impact of amoxicillin prophylaxis on single-tooth extraction.	Amoxicillin has a significant impact on bacteremia resulting from a single-tooth extraction. Toothbrushing may be a greater threat for individuals at risk for infective endocarditis.
Mancini et al. (2018) Virulence [49]	Animal (Switzerland pilot)	Rat with catheter-induced aortic vegetations	To investigate the role of Coa and vWbp in IE initiation	Coa does not support the initial colonization of IE (in *L. lactis*). vWbp contributes to the initiation of IE (in *L. lactis*) however is marginal in the presence of ClfA.
Reguiero et al. (2019) Circ. Cardiovasc. Interv. [51]	Human Comparative Multicenter (Canada pilot)	245 pts SEV 115 vs. BEV 130	To determine the incidence, clinical characteristics, and outcomes of patients with IE post-TAVR	IE post-TAVR did not reveal early or late mortality
Rodríguez-Vidigal et al. (2019) Enferm. Infecc. Microbiol. Clin. [52]	Human Observational Retrospective (Spain)	200 pts with TAVI	To evaluate single-centre experience of incidence, mortality, and associated factors of IE after TAVI.	Incidence of IE post-TAVI greater than other series.
Di Carluccio et al. (2021) RSC Chem. Biol. [55]	Human Multicenter (Italy pilot)	Collected anatomical specimen	To evaluate the mechanism of interaction of SLBR-B and SLBR-H from *S. gordonii* in causing IE	Streptococcal Siglec-like adhesins spark the development of tailored synthetic inhibitors and therapeutics specific for Streptococcal adhesins to counteract IE. No impairment of the interplay between Siglecs and glycans.
Manukumar et al. (2017) Sci. Rep. [56]	Human Single Center (India)	Collected blood draws	To characterize MRSA strain using MALDI-Biotyper multiplex PCR to distinguish between MRSA and MSSA. To screen PCR-SSCP	PCR-SSCP technique for rapid detection of MSSA and MRSA strains was developed
Mempel et al. (2002) Br. J. Dermatol. [57]	Human Single Center (Germany)	^†^ *S. aureus* DU 5720 vs. *S. aureus* DU 8325-4 vs. *S. aureus* DU 5883	To investigate haemolysin-independent virulence in human keratinocytes.	Staphylococcal invasion of human keratinocytes independently of alpha- and beta-hemolysins, leads to necrotic and apoptotic cell damage.
Nakagawa et al. (2017) Cell Host Microbe J. [58]	Animal Multicenter Center (Japan pilot)	Murine epicutaneous infection model	To evaluate how *S. aureus* trigger inflammation	Increased production of IL-1α, IL-36α and Il 17 via IL-1R and IL-36R. Increased γδ T cells, ILC3 and neutrophil. Keratinocyte * Myd88 signaling in response to *S. aureus* PSMα drives an IL-17-mediated skin inflammatory response to epicutaneous *S. aureus* infection.
Schwarz et al. (2021) Virulence [63]	Human in vitro and in vivo Multicenter (Germany)	34 *S. aureus* Pts with *S. aureus* endocarditis vs. healthy individuals	To evaluate pathomechanisms in the induction of IE	in vitro assays did not correlate with the severity of IE. *S. aureus* isolates differed in the activation and inhibition of pathways connected to the extracellular matrix and inflammatory response
Malachowa et al. (2011) PLoS ONE [64]	Human/Animal Single center (USA)	*S. aureus* LAC vs. *S. aureus* LACΔhlgABC	To study the *S. aureus* USA300 transcriptome	Limited contribution of any single two-component leukotoxin lukS-PV and lukF-PV to USA300 immune evasion and virulence.
Alonso et al. (2013) Nature [65]	Animal Single center (USA)	CCR5-deficient mice	To study activity of *S. aureus* leukotoxin ED (LukED)	CCR5-deficient mice are resistant to lethal *S. aureus* infection
Kim et al. (2010) J. Exp. Med. [71]	Animal Single center (USA)	λ Mice with SpA (KKAA)	To study *S. aureus* protective immunity.	SpA (KKAA) immunization enabled MRSA-challenged mice to organize antibody responses to many different staphylococcal antigens.
Becker et al. (2014) Proc. Natl. Acad. Sci. USA [72]	In vitro Single center (USA)	*S*. *aureus* Newman cultures	To demonstrate that SpA is released with murein tetrapeptide-tetraglycyl [L-Ala-D-iGln-(SpA-Gly5) L-Lys-D-Ala-Gly4] linked to its C-terminal threonyl	SpA, a B cell superantigen, is released with peptidoglycan linked to its C terminus. Murein hydrolases cleave the anchor structure of released SpA to modify host immune responses.
Zhang et al. (2015) Infect. Immun. [84]	Animal Single center (China)	Mice SaEsxA and SaEsxB vs. Mice rSaEsxA and rSaEsxB	To investigate SaEsxA and SaEsxB, as possible targets for a vaccine.	SaEsxA and SaEsxB are effective toward Th1 and Th17 candidate antigens.
Brady et al. (2013) PLoS ONE [85]	Animal Single center (USA)	Mice HlaH35L vs. Control vs. Prosthetic implant model of chronic biofilm	To evaluate the ability of one *S. aureus* vaccine antigen to protect in three mouse models of infection	Vaccines may confer protection against one form of *S. aureus* disease without conferring protection against other disease presentations
Zhang et al. (2018) mBio [86]	Animal Multicenter (USA pilot)	C57BL/6 mice	To study the role of adaptive immunity induced by an *S. aureus* vaccine in protection against *S. aureus* bacteremia	Multipronged humoral and cellular (B-cell, Th1, Th17) responses to *S. aureus* antigens may be critical to achieve effective and comprehensive immune defense
Yu et al. (2018) Sci. Rep. [87]	Animal Single center (China)	Mouse peritonitis model	To evaluate the humoral immune response and CD4^+^ T cell-mediated immune responses	The MntC-specific antibodies and MntC-specific Th17 cells play cooperative roles in the prevention of *S. aureus* infection.

Abbreviations: BEV, balloon-expandable valve; C57BL/6, C57 black 6; CCR5, C-C chemokine receptor type 5; ClfA, clumping factor A; Coa, plasma-clotting factors staphylocoagulase; DU, *S. aureus* mutant; IE, infective endocarditis; γδ T cells, Gamma delta T cells; IL, interleukine; ILC3, group 3 innate lymphoid cells; HaCaT, aneuploid immortal keratinocyte cell; LAC, wild-type USA300 strain; LACΔhlgABC, hlgABC-deletion strain; *L. lactis*, *Lactococcus lactis*; lukS/F-PV, leukotoxin S/F-Panton-Valentine; LukED, *S. aureus* leukotoxin ED; MntC, *S. aureus* manganese transport protein C; MRSA, methicillin-resistant *Staphylococcus aureus*; MSSA, methicillin-susceptible *S. aureus*; PCR, protein chain reaction; PCR-SSCP, PCR-coupled single strand conformation polymorphism; PSM, phenol-soluble modulin α; Pt, patient; PVL, Panton-Valentine Leukocidin; rSaEsx, recombinant; SaEsx, *S. aureus* Esx; SEV, self-expanding valve; SLBR, Siglec-like binding region; SpA, staphylococcal protein A; TAVI, transcatheter aortic valve implantation; T_h_17, T helper 17 cells; TSB, trypticase soy broth. ^†^ *S. aureus* mutant DU 5720 alpha-haemolysin, beta-haemolysin double-negative; *S. aureus* mutant virulent strain DU 8325-4; *S. aureus* variant DU 5883 isogenic fibronectin-binding protein A/B-negative. * Myd88, keratinocyte-specific deletion of the IL-1R and IL-36R; λ variant KKAA staphylococcal protein A.

### 3.5. Biofilm Formation

Biofilms allow pathogens to live by conforming to the functions and metabolism of the self-produced matrix, which is composed of hydrated extracellular polymeric substances (EPS). Therefore, biofilms behave as an immediate functional environment constituted directly by the bacteria. The primary constituents that organize EPS are molecules of polysaccharides, proteins, nucleic acids, and lipids. EPS performs varied functions involving the conferral of mechanical stability of biofilms. Furthermore, EPS mediates the adhesion of bacteria to surfaces by forming a cohesive and three-dimensional polymer network that interconnects and transiently immobilizes the biofilm cells. The external digestive system of the biofilm matrix keeps extracellular enzymes close to the cells, which can be metabolized and dissolved into colloidal and solid biopolymers [82,88,89].

During the course of infective endocarditis, the production of bacterial biofilms is a basic phase for the fatal evolution of the disease. IE manifests itself as a lesion of the cardiac structure and causes a healing reaction, which advocates the recruitment of fibrin and immune cells. In the first cicatricial stage, the vegetation is sterile but potentially at risk of causing colonization over temporary bacteremia, thus promoting well-established IE. In vitro, experimental models using a simulated IE vegetation model, produced from venous whole blood, have been demonstrated to be of great utility for assessing biofilm generation in infective endocarditis. Similarly, these models allowed for the establishment of stable bacterial colonization after 24 h. Once organized in biofilm aggregates, the pathogens revealed higher tolerance to antibiotics [88,89].

Swartz and colleagues recently studied the momentum required to produce biofilms and how these affect the maturing of antibiotic tolerance. Evidence noted that reference strains of *Staphylococcus aureus* as well as three clinical cases of IE produced biofilms modeled on IE vegetation six hours after the onset of infection. Thus, the earlier the antibiotics were administered, the more marked their pharmacological action in containing biofilm maturation, indicating early treatment was more effective in restraining the spread of the disease. The investigators followed the biofilm development under the microscope by observing the bacterial aggregates growing on the IE vegetation model and the interaction with the antibiotic. The generation of mature, antibiotic-resistant biofilms were recorded six hours later, thus precipitating screening for optimal treatment strategies for IE [90].

Biofilm formation raises concerns in patients requiring the treatment of heart valve endocarditis (HVE) [91,92,93,94]. In this context, the aggressiveness of Gram-positive bacteria becomes crucial due to the lack of an external membrane that is replaced by the surrounding peptidoglycan, less sensitive to serum-induced killing. Subsequently, to bacteria colonization and adhesion, the pathophysiology of HVE is characterized by bacterial proliferation cycles. In this phase, local thrombotic processes, the recruitment of monocytes, and inflammation lead to the formation of mature vegetations occur [50]. Regarding HVE, the production of biofilm is representative of numerous causative pathogens, including staphylococci, streptococci, and enterococci with other rarer organisms, such as *Pseudomonas aeruginosa* and *Candida* species, that promote bacterial incorporation into a polysaccharide extracellular slime-like matrix. In patients with staphylococcal prosthetic valve endocarditis (PVE), undergoing valve replacement with the use of a homograft or autograft [91,92,93,94], the specificity of biofilms induces a cell-to-cell communication and synchronized gene expression that promotes the assembly and maturation of pathogens. In this population of patients, once the biofilm arises, it protects the bacteria from the host’s immune system and reduces antimicrobial efficacy while shielding the organisms [50].

The characteristics of the generating biofilm are now recognized as virulent traits in the development of PVE, especially when related to *Staphylococcus aureus*, for which the use of allogeneic or autologous tissue as an ideal valve substitute is recommended. Cryopreserved Aortic Homograft (CAH) is widely used in prosthetic valve endocarditis (58.1% vs. 28.8%, *p* = 0.002) and methicillin-resistant *Staphylococcus* infection (25.6% vs. 12.1%, *p* = 0.002), compared to patients with conventional prostheses [95]. In another report, 64% of patients with PVE involving the aortic valve received an aortic homograft in 56 (64%) patients while mechanical prosthesis was used in 23% of cases and a bioprosthetic in 13%, respectively. Surgical correction using an aortic homograft was independently associated with a reduced risk of infection relapse (*p* = 0.006) compared to conventional valves [96]. Active endocarditis supported by causative pathogens generating biofilm is often responsible for recurrence [97,98,99,100,101] and is a statistically significant univariable risk factor for increased early and late mortality as revealed by studies with short- [95,100] and long-term follow-up (over 20 years) [102,103,104,105,106,107]. As far as PVE is concerned, the use of CAH appears indisputable, unlike native valve endocarditis whereby the preference for conventional prosthesis and synthetic material is still prevailing [96].

We used cryopreserved aortic homograft as a substitute to replace aortic and mitral valve diseases in 56.2% and 21% of patients, in which abscess formation occurred. The process was sustained by causative pathogen-generating biofilm and resistance to antibiotic treatment [18,98,101,102,104,107]. Sometimes, in the presence of aggressive IE with an extension to the aorto–mitral junction and mitral valve, we used a double homograft valve implant [18,102,107,108,109,110,111,112]. During the cryopreservation process, the homograft was processed in combination with the application of antibiotics (gentamicin, vancomycin, metronidazole, piperacillin, flucloxacillin, tobramycin, meropenem, colistin, and antifungal amphotericin B), which promoted a significant influence on the resistance of the allogeneic tissue to infections. Ascending aortic homograft tissue revealed significantly improved resistance against *S. epidermidis* and *S. aureus* with a lower propensity for bacterial contamination than homograft aortic valves. For the latter, the highest risk of bacterial biofilm formation persists, especially induced by *Staphylococcus aureus*, which is difficult to penetrate. Along the same lines, more effective resistance was observed against *P. aeruginosa* using flucloxacillin and *E. coli* using meropenem and colistin [113] (Table 2).

**Table 2:** Characteristics of the Included Studies.

First Author/Year/Ref	Type of Study	Cohort	Aims	Finding
Schwartz et al. (2021) APMIS [88]	In vitro patch enriched with platelet and Leucocyte-rich fibrin Multicenter (Danemark)	IE organoid-like model by colonization with IE-associated bacterial isolates *S*. *aureus*, *S. mitis* and *Enterococcus faecalis* (IE vegetation (IEV)	To establish an in vitro vegetation simulation IE model for fast screening of novel treatment strategies	The surface-associated bacteria displayed increased tolerance to antibiotics compared to planktonic bacteria. IE simulation model with the relevant pathogens *S. aureus*, *S. mitis* group, and *E. faecalis* was established and IE model mirrors the natural IE process
Di Domenico et al. (2019) BMC Microbiol. [89]	Human Multicenter (IT)	Samples of infected heart tissue. *S. aureus* 50%, *Enterococcus faecalis* 25% and *Streptococcus gallolyticu*s 25%	To assess a rapid biofilm identification assay and a targeted antimicrobial susceptibility profile of biofilm-growing bacteria in patients with IE, which were unresponsive to antibiotic therapy	Biofilm-producing bacteria, from surgically treated IE, display a high tolerance to antibiotics, which is undetected by conventional antibiograms
Schwartz et al. (2012) APMIS [90]	Animal model Multicenter (Danemark)	IE organoid-like model by colonization with IE-associated bacterial isolates *S*. *aureus*, *S. mitis* and *Enterococcus faecalis* (IEV)	To evaluate the time course of biofilm formation and the impact on antibiotic tolerance development	The antibiotic effect was significantly higher than when treatment was started after the biofilm was allowed to mature
Kim et al. (2016) JTCVS [95]	Human Single Center (USA)	86 pts Homografts vs. 139 pts Xenograft prostheses vs. 79 pts Mechanical prostheses	To evaluate resistance to infection	Homografts were more used in PVE (*p* = 0.002) and methicillin-resistant *Staphylococcus* (*p* = 0.002), compared with conventional prostheses. No significant benefit to the use of homografts was demonstrable with regard to resistance to reinfection in the setting of IE
Nappi et al. (2018) JTCVS [102]	Human Single center (France)	210 pts	To evaluate long-term results of aortic allografts and to identify factors influencing long-term durability	The use of allograft is a valid option in complex infective endocarditis and in women of childbearing age
Steffen et al. (2016) JTCVS [113]	In vitro Single center (Germany)	10 cryopreserved human allografts	To evaluate the in vitro antimicrobial activity of 3 antibiotic regimens	Allograft antibacterial activity despite long-term storage over 5 years. Antibiotic combinations applied during CHA processing have a significant influence on their infection resistance. Ascending aortic tissue shows a significantly enhanced bacterial resistance against staphylococcal bacteria compared with aortic valves

Abbreviations: IEV, infective endocarditis vegetation; pts, patients; PVE, prosthetic valve endocarditis; *S*. *aureus*, *Staphylococcus aureus*; *S. mitis*, *Streptococcus mitis*.

### 3.6. Interaction of Staphylococcus aureus with Coagulation Mechanisms

*Staphylococcus aureus* infections have been extensively studied with the use of different animal models specially adapted to invasive infections of this pathogen, suggesting the fundamental role of two coagulases, von Willebrand factor binding protein (vWbp) and coagulase (Coa), which account for its virulence. These molecules form a functionally intricate architecture that *S. aureus* uses to generate a protective fibrinogen/fibrin shield that surrounds it. The emergence of this armor yields the pathogen the potential to circumvent the defense system implemented by the host’s phagocytic cells. One of the pivotal functions of coagulases promotes the non-proteolytic activation of the zymogen pro-thrombin to transform fibrinogen into fibrin, thus contributing to the emergence of the fibrinogen/fibrin safeguarding shield.

**Figure 4:**
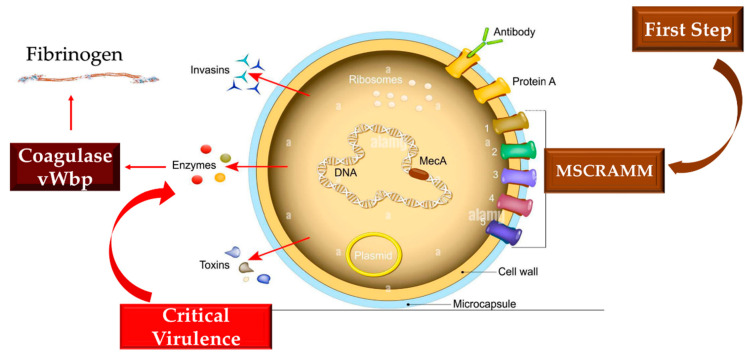
Virulent factors of *S. aureus* are reported. MSCRAMMs drive with a substantial key role in the initiation of endovascular, bone, and joint, alongside prosthetic-device infections. These structures can bind to molecules such as collagen (mostly via Cna), fibronectin (via FnbAB), and fibrinogen (with ClfAB and Fib) and thus evade the immune system. The development of infective process is induced by Coa and von Willebrand factor binding protein that led to critical virulence. Coa binds preferentially soluble fibrinogen while vWbp did not disclose any preference across the two forms of fibrinogen. Abbreviations: Coa, coagulase; MSCRAMM, microbial surface components recognizing adhesive matrix molecules; vWbp, von Willebrand factor binding protein. Permission from [12], https://doi.org/10.3390/metabo12080682.

There are many essential functions of coagulases. One of these influences the non-proteolytic activation of the prothrombin zymogen to convert fibrinogen to fibrin, thus leading to the genesis of the protective fibrinogen/fibrin shield. Another function promoted by coagulases is to serve as a linkage with fibrinogen, whose interactions greatly sustain infection. The mechanism or mechanisms that enable the binding between vWbp and Coa and fibrinogen entail well-defined interactions of the two proteins with the molecule, although they show a similar structure. Coa binding to soluble fibrinogen has a significantly higher affinity than fibrinogen coated on a plastic surface. The vWbp, on the other hand, did not show any preference between the two forms of fibrinogen [10,11,12,13,14] (Figure 4).

Thomas and colleagues investigated the complex interactions between fibrinogen and *S. aureus*, suggesting a different action exerted by vWbp and Coa targeting different sites on fibrinogen, demonstrating an absence of conflict among the two molecules in fibrinogen binding. Both Coa and vWbp have N- and C-terminal halves that drive fibrinogen binding activity [13,14]. These vWbp coagulases have higher fibrinogen binding affinity in the vWbp-N region in divergence to Coa, in which the major bias towards the fibrinogen binding site has been related to the C-terminal region. It has been observed that the peptides constituting the formerly recognized Fibrinogen Coa/Efb1 binding motif do not impede the vWbp-C constituent from attaching to fibrinogen. Therefore, non-attendance of a functional homolog to this motif has been suggested for vWbp-C. It was also observed that although the N-terminal prothrombin-binding domains of both coagulases recognize the β-chain of fibrinogen, they nevertheless seem to interrelate with several sequence motifs in the host protein. It is therefore possible to speculate that the interplay of the two coagulases seems to be exhibited with divergent sequence motifs in the host protein. The findings reported by Thomas et al. give new awareness to the intricate interlinkage among Fg and *S. aureus* coagulases [14].

Multidrug-resistant *S. aureus* strains are accountable for life-threatening diseases deploying a worldwide public health concern. The restrictions for dealing with *S. aureus* infection rely on both the treatment and the absence of a fruitful vaccine. As formerly indicated, *S. aureus* develops complex and errorless mechanisms that preserve it through the protection of a shield by fibrinogen/fibrin. This coating serves two objectives: (1) it permits the bacterium to survive in the blood rendering it invisible to the host’s immune protection and (2) it provides the likelihood of spreading and giving rise to invasive diseases. Modifying this process depicts an encouraging aim for new antistaphylococcal treatment strategies; however, the mechanisms that adjust the phenomena are not yet entirely inquired. *S. aureus* expresses many proteins that tie to fibrinogen. A redundant action exerted by some of these molecules with vWbp can limit its function. Sharing between proteins expressing similar functions in the structural or functional motif has often been suggested.

Thomas and colleagues [14] argued the expression of a protein homolog vhp corresponding to the C-terminus of the von Willebrand factor binding protein (vWbp) contributes to shield assembly and fibrinogen binding. They recognized a common Fg binding motif between vhp and vWbp.

Recently, Schwartz and colleagues [63] illustrated the potential pathomechanisms using both in vitro and in vivo models of 34 isolates of *Staphylococcus aureus*, which were evaluated by gathering causative pathogens from patients with *S. aureus* endocarditis and healthy subjects. The strains of *S. aureus* isolated were assessed in vitro to analyze cytotoxicity and the invasion and interrelation with platelets typically revealed by these bacteria. In order to correlate the faculty of *S. aureus* to advocate the development of vegetations on the aortic valves in vivo, the virulence factor expression profiles and cellular response were also assessed using an animal model. The existence of IE involving valves was evaluated in vivo with the use of magnetic resonance imaging at 9.4 T. A histological assessment with enrichment gene expression analysis was also fulfilled. *S. aureus* isolated and investigated in vivo revealed the potential of causing IE by reliably inducing inflammatory responses associated with the aortic valve’s injuries. However, the differentiation and classification of IE as well as the characterization of inflammation based on the measurement of in vitro virulence profiles and cytotoxicity was not established [63].

Schwartz and colleagues [63] observed that in vitro test results did not correlate with IE severity. However, the researchers noted that the *Staphylococcus* isolates differed considerably in the degree of activation and inhibition of pathoanatomical processes related to the extracellular matrix and in the features of the inflammatory reaction. It was therefore suggested that the pathogenic ability of the pathogen did not bestow a constant response and that more comprehensive approaches to host–pathogen interactions were required for its assessment. Furthermore, this approach promoted new insights into the corresponding immune pathways to highlight differences in host–pathogen interactions [63].

With regards to the etiology of *S. aureus*-promoted infective endocarditis, Schwarz and colleagues [63] permitted better comprehension on the interaction between virulence factors and immune responses in *S. aureus*-borne infective endocarditis, thus promoting the spread of innovative therapeutic strategies and specific diagnostic imaging markers.

### 3.7. Involvement of Vascular Endothelium and Blood Constituents in S. aureus-Induced Endocarditis

*Staphylococcus aureus* surface molecules operate crucially to favor the colonization of the vascular endothelium, which is a pivotal primary event in the pathogenesis of IE. The faculty of these molecules to elicit associated endothelial procoagulant and proinflammatory responses, promoting the progress of infective endocarditis, has been well-stated [73,114,115,116,117]. Heying and colleagues [114] assessed the peculiar role of three fundamental molecules expressed on the surface of *S. aureus*. Fibronectin-binding protein A (FnBPA) and B (FnBPB) as well as clumping factor A (ClfA) act to promote bacterial adherence that identifies the cultured human endothelial cells (ECs) interacting with *S. aureus*. Likewise, these molecules encourage phenotypic and functional modifications in ECs. The investigators used a non-invasive surrogate bacterium *Lactococcus lactis*. *Lactococcus lactis*, by gene transfer, expressed staphylococcal molecules FnBPA, FnBPB, or ClfA. In this way, the recombinant Lactococci positive for FnBPA or FnBPB revealed an increase in the incidence of infection at the EC level by up to 50–100 times the baseline threshold. Other evidence highlighted the provocation of an inflammatory response with the activation of the EC characterized by an increased expression of ICAM-1 and VCAM-1 on the surface and the production of interleukin-8 associated with the concomitant adhesion of monocytes. On the contrary, an infection determined by ClfA-positive lactococci did not activate the EC. The leading action of FnBPA-positive *L. lactis* promoted a notable inflammatory response that was enhanced by cell-bound monocytes and mediated by tissue factor-dependent endothelial coagulation. Evidence suggested that *S. aureus* FnBPs, but not ClfA, promoted the invasiveness and pathogenicity of nonpathogenic *L. lactis* microorganisms, pointing out that bacterium–EC interactions mediated by these adhesins were strongly inclined to promote coagulation and inflammation in infected endovascular sites [114].

Studies carried out in experimental endocarditis induced by *Staphylococcus aureus* have highlighted two important phases of the infection. The purpose of sequential fibrinogen binding in charge of valve colonization and the pivotal role of fibronectin-binding promoting endothelial invasion was demonstrated. These biological phenomena were supported by peptidoglycan-linked adhesins. The function played by fibronectin-binding protein A (FnBPA) promoted a combination of these two determined properties, merged with the binding of elastin, in favoring experimental endocarditis.

**Figure 5:**
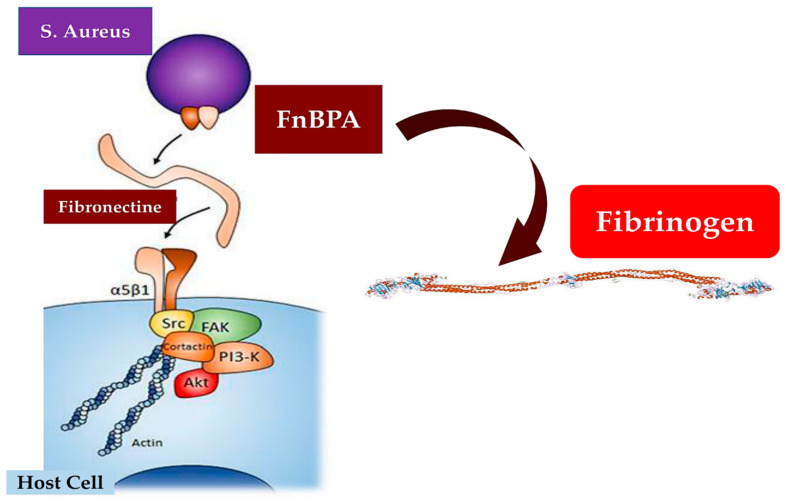
Depict the mechanism of experimental endocarditis caused by *S. aureus*. The process is marked by the crucial role of sequential fibrinogen binding responsible for valve colonization and the critical action of fibronectin-binding that promotes endothelial invasion. FnBPA responsible for fibrinogen and fibronectin binding may advocate cell invasion in vivo endocarditis. Abbreviations; Akt or PKB, protein kinase B, FAK, focal adhesion kinase; P13-k, Phosphoinositide 3-kinase; Src, proto-oncogene tyrosine-protein kinase. Permission from [12], https://doi.org/10.3390/metabo12080682.

One study reported the substantial role played by the minimal sub-domain of FnBPA accountable for fibrinogen and fibronectin binding in promoting cell invasion in endocarditis in vivo. FnBPA was expressed in *Lactococcus lactis* and was assessed in animal models and in vitro [115]. The subdomain needed to induce IE comprised 127 amino acids that depicted the hub of the fibrinogen- and fibronectin-binding regions of FnBPA and were adequate to bestow a charge to these assets. Although in animals evidence noted the crucial role of fibrinogen binding to determine endocarditis induction, the role exerted by fibronectin binding was not significantly coupled with endocarditis development. Instead, as for disease acuteness, both fibrinogen binding and fibronectin binding were of substantial importance. Besides, the synergistic merger of fibrinogen binding and fibronectin binding suggested a considerable enhancement in the infectious foray of cultured cell lines, emphasizing a decisive feature linked with the severity of endocarditis. Accordingly, the concept based on sequential action offered by fibrinogen binding and fibronectin binding in fostering colonization and invasion could be used for the development of anti-adhesin strategies [115] (Figure 5).

Bacterial proteins, such ClfA and FnBPA, intercede for the adhesion of *S aureus* to EC surface molecules. This purpose is shared with subendothelial matrix proteins involving fibrinogen, fibrin, fibronectin, and von Willebrand factor (vWF) [116]. It is important to underline the work of Pappelbaum et al. [117], who suggested ultra-large von Willebrand factors (ULVWF) substantially concurred with the inceptive pathogenic step of *S. aureus*-induced endocarditis in subjects with healthy untouched endothelium. The synergistic role of ClfA, FnBPA, and von Willebrand factors (vWF) in determining the adhesion of *Staphylococcus aureus* to endothelial cells (ECs) has been investigated in three recent reports that markedly endorse the fundamental importance of these molecules in IE [118,119,120]. Evidence pointed out that ultra-large von Willebrand factors (ULVWF) substantially promoted the initial pathogenic phase of *S. aureus*-induced endocarditis in patients with undamaged endothelium. The use of heparin and ADAMTS13 reduced ULVWF formation and may serve as a novel therapeutic choice to avoid IE [117].

Recently, Claes et colleagues [118] revealed the interaction between vWbp and proteins expressed on the surface of *S. aureus* that moderated the bacterium adhesion to VWF and to vascular endothelium under shear stress. Mutants deficient in Sortase A (SrtA) and SrtA-dependent surface proteins, as well as *Lactococcus lactis* transmitting single staphylococcal surface proteins, have been used. In detail, *S. aureus* first attached to the endothelium via vWF, raising levels of the VWF-binding protein (vWbp) that finalized the adhesion of *S. aureus* to VWF under shear stress, and lastly, the vWbp interconnected with vWF and the Sortase, a ClfA dependent surface protein. Therefore, it is possible to affirm that vWF-vWbp-ClfA anchored *S. aureus* to the vascular endothelium under shear stress [92]. In another report, the same investigators studied the effect of shear flow and plasma on the binding of ClfA and FnBPA, comprising its sub-domains A, A16+, ABC, CD, vWF, fibrinogen/fibrin, fibronectin, or confluent ECs. With the use of a genetically engineered *Lactococcus lactis* that exhibited these adhesins heterologously, Claes et al. [119] found that comprehensive adherence profiles were almost alike in static and flow conditions. The level of adhesion of *L. lactis*-FnBPA to EC-bound fibronectin and of *L. lactis*-ClfA to EC-bound fibrinogen was similar to that of L. lactis-ClfA to coated vWF domain A1 in the presence of vWF-binding protein (vWbp). Thus, in plasma, the adhesion of *L. lactis*-ClfA to activated EC-vWF/vWbp was reduced by 80% within the time limit of 10 min, and this event was associated with the paramount role of disintegrin-mediated and metalloproteinase-mediated vWF hydrolysis with thrombospondin motif type 1, member 13. Equally, in lacking plasma components, the adhesion of *L. lactis* FnBPA was decreased by >70%. Instead, plasma fibrinogen noted a high binding affinity of *L. lactis*-ClfA to resting and activated ECs. These findings suggest that in plasma, *S. aureus* adhesion to active endothelium was dependent mostly on two supportive pathways: a rapid but short-lived vWF/vWbp pathway and a stable integrin-coupled–fibrinogen pathway. Observations derived from these findings suggest that the pharmacological inhibition of ClfA–fibrinogen interactions may play a role in the adjunctive treatment of infective endocarditis [119].

*Staphylococcus aureus* actively invades the endothelium, promoting detrimental action that causes apoptosis and endothelial damage. The literature supports the knowledge of the crucial role of *Staphylococcus* in causing IE by protein clumping factor A (ClfA), which interconnects to the cell wall of *S. aureus*. Several reports have recently elucidated mechanisms of secreted plasma coagulation factors staphylocagulase (Coa) and the protein binding von Willebrand factor (vWbp). Mancini et colleagues [49] assessed rat models with catheter-induced aortic vegetation. They studied the function of staphylococcal secreted coagulase (Coa-positive staphylococci) and *Staphylococcus aureus* encoding the von Willebrand factor binding protein (vWbp) in the development of IE. As previously reported, a model based on *Lactococcus lactis* mutants expressing coa, vWbp, ClfA, or vWbp/clfA and *S. aureus* Newman Δcoa, ΔvWbp, ΔclfA, or Δcoa/ΔvWbp/ΔclfA was used. The investigators noted that vWbp expression statistically raised *L. lactis*-induced valve infection in contrast to strains expressing coa. Likewise, ClfA expression revealed an increase in the infectiousness produced by *L. lactis*, which was not further affected by vWbp co-expression. Of note the finding that effacement of Coa or vWbp genes in *S. aureus* did not reduce infectivity whilst annulment of ClfA function dramatically diminished valve infection. A decisive observation advocated that the function of clfA was not influenced by the triple deletion of Δcoa/ΔvWbp/ΔclfA. This result allowed hypothesizing that Coa did not promote colonization of inceptive IE using *L. lactis* as a pathogen in the absence of other key virulence factors. The presence of vWbp concurred with the onset of IE induced by *L. lactis*, but its role was borderline in the attendance of ClfA [49].

Although evidence has shown the pathogenic extracellular role of *Staphylococcus aureus*, this causative pathogen also can be integrated by host cells, including non-specific phagocytes. Therefore, it can be a deterrent to endothelial cells, epithelial cells, or osteoblasts. The intracellular *S. aureus* location concurs with the establishment of the infection. The entry gate of the bacterium is umpired by the binding of integrin α5β1 expressed on the membrane of the host cell, which recognizes fibronectin. This bridge encourages the recognition between pathogen and host cell promoting subsequent cell integration [121,122,123,124]. Although the osteoblasts revealed a tall expression of α5β1-integrin and fibronectin with demonstrable adherence to osteoblasts, Niemann and colleagues [125] suggested, using internalization tests and immunofluorescence microscopy, that *S. aureus* was less engulfed by osteoblasts compared to epithelial cells. The authors noted that throughout the cell infection, adding exogenous fibronectin in the presence of *S. aureus* increased uptake of the pathogen in epithelial cells that was not disclosed in osteoblasts. This evidence offered understandable contrast to prior claims concerning the pathogen uptake mechanism, which yielded integrin and fibronectin expression, a pivotal action in causing bacterial uptake in host cells. Importantly, the arrangement of extracellular fibronectin surrounding osteoblasts and epithelial cells was dissimilar, revealing a typically structured frame of a fibrillar network in the former. The uptake enhancement of *S. aureus* was significant, arising from the inhibition of fibril production, brief lowering of RNA-mediated fibronectin expression, and disruption of the fibronectin–fibril network. The study of Nieman and colleagues [125] demonstrated that the fibronectin–fibril network reduced the uptake of *S. aureus* into a given host cell, suggesting that the supramolecular structure of fibronectin may govern the dissimilar ability of peculiar host cells to internalize the bacterium. The evidence reported by Niemann et al. [125] advocated the non-determining function deployed by the crude amount of fibronectin and the unfavorable function denoted by the supramolecular structure of the fibronectin molecules.

Once stored on the eukaryotic cell surface, they exert a fundamental action in bacterial uptake by the host cells. This evidence may describe the remarkable inconsistency expressed in the efficiency of *S. aureus* uptake by various host cell types. In addition, in vivo discrepancies between bacterial infection courses and bacterial localization have been demonstrated in different clinical settings [125].

From a molecular point of view, the pathogenicity of *S. aureus* is linked to the expression of virulence factors, comprising proteins that moderate the process of adhesion to host plasma molecules and extracellular matrix proteins. Among these, numerous shreds of findings have demonstrated a marked ability of IsdB-expressing bacteria to adhere to both soluble and immobilized vWF [126]. A recent study by Alfeo et al. [127] highlighted that the iron-regulated surface determinant B (IsdB) protein, besides iron transport and vitronectin binding, interacted with the von Willebrand factor (vWF). The binding between IsdN and recombinant vWF was disrupted by heparin and was also reduced due to the high ionic strength. Thus, the use of administered ristocetin, an allosteric agent that induced the exhibition of the A1 domain of vWF, elicited the considerable effect of rising the binding between IsdB and vWF. It was permissible to speculate that IsdB-binding and *S. aureus* adhesion were markedly impeded by a monoclonal antibody averse to the A1 domain as well as IsdB reactive IgG isolated from subjects with staphylococcal endocarditis. This evidence suggested two obvious conclusions: the importance of IsdB in promoting *S. aureus* adhesion and its role in the colonization of the endothelium. Again, the potential role of IsdB as a therapeutic target could be offered [127]. Recently Nishitani and colleagues [128] suggested that IsdB-immunized CD163−/− mice were resistant to sepsis following *S. aureus* SSI, as were normal healthy mice given anti-CD163-neutralizing antibodies. These genetic and biological CD163 deficiencies did not exacerbate local infections. Thus, anti-IsdB antibodies are a risk factor for *S. aureus* sepsis following SSI, and disruption of the multimolecular complex and/or CD163 blockade may intervene. This study was timely before that of Tsai and colleagues who evaluated the non-protective immune imprint underlying the failure of the *Staphylococcus aureus* IsdB vaccine. Vaccine interference was overcome by immunization against the IsdB heme-binding domain. Purified human IsdB-specific antibodies also blunt the IsdB passive immunization, and additional SA vaccines are susceptible to SA pre-exposure. Thus, failed anti-SA immunization trials could be explained by non-protective imprint from prior host–SA interaction [128,129].

### 3.8. Infective Endocarditis and Platelets

In patients at an increased risk of infective endocarditis, the use of antibiotic prophylaxis is currently recommended, given the difficulty in treating IE and its inherent mortality. It should be underlined that the concerns correlated with the administration of antibiotics are faced with their unquestionable low efficacy for certain strains of *Staphylococcus aureus* alongside the perpetuation of increasing multidrug-resistant strains of infection. Given this worrying clinical scenario, the need to discover new therapeutic options remains a priority against IE. The role played by platelets is decisive in the early phase of infective endocarditis, making them first-line immune responders [75,76,130].

Important results have been observed in mechanistic in vitro studies, which have highlighted the early action of the infection supported by platelets during the first phase of the *S. aureus* infection involving cardiac structures. The first front of the platelet-dependent immune response can be configured in directing an initial antimicrobial contrast action mediated by the interaction of platelets with the pathogen. This is the proposed case for the therapeutic use of acetylsalicylic acid.

Several experimental and clinical reports have suggested that the purpose of aspirin may restrict bacterial–platelet interactions promoting the prevention of vegetation spread and have revealed promising results. However, the data from clinical trials reporting outcomes in patients with IE who received additional aspirin to background therapy have not produced conclusive results. Therefore, conflicting evidence emerged, shedding a veil of uncertainty about the advantage of antiplatelet drugs in the prevention of IE sustained by *S. aureus*. In addition to aspirin, other drugs with anti-platelet actions have also been tested, for which a therapeutic effect has been observed. For example, the P2Y12 platelet receptor antagonist ticagrelor could couple its potent and well-known antiplatelet role with noticeable antibacterial properties. Furthermore, a recent study based on a mouse animal model reported a pronounced capacity of ticagrelor to eradicate *S. aureus* bacteremia [131,132,133,134,135] (Table 3).

**Table 3:** Characteristics of the Included Studies.

First Author/Year/Ref	Type of Study	Cohort	Aims	Finding
Que et al. (2005) J. Exp. Med. [73]	Animal model Single Center (Switzerland)	Rat model of IE induced	To study valve colonization with experimental endocarditis. To evaluate the role of ClfA and FnBPA positive lactococci	Fibrinogen and fibronectin binding could cooperate for S. aureus valve colonization and endothelial invasion in vivo
Edwards et al. (2012) PLoS ONE [74]	Human Single Center (UK)	Blood sample	To study in vivo role of Eap to interact with host glyco-proteins	Eap expressing strains cause a more severe infection, demonstrating its role in invasive disease. Increased level of TNFα and gC1qR/p33 expression
Veloso et al. (2013) Infect. Immun. [76]	Animal model Single Center (Switzerland)	Rat model of induced IE 10(6) CFU *L. lactis* pIL253 vs. Recombinant *L. lactis* (ClfA, FnbpA, BCD, or SdrE)	To explore the contributions of *S. aureus* virulence factors to the initiation of IE	Fibrinogen binding in the initiation of *S. aureus* IE. Activation of platelet aggregation or an inflammatory response may contribute to or promote the development of EI
Thomas et al. (2021) mBio [77]	Animal model Single Center (USA)	Rat model of IE induced	To identify proteins with significant amino acid identities to vWbp	Protein homologous to the C-terminal of vWbp was identified. Its role in Fg shield assembly and binds
Hussain et al. (2002) Infect. Immun. [78]	In vitro Single center (Germany)	*S*. *aureus* Newman cultures vs. Control mutant	To investigate the role of Eap by constructing a stable eap::ermB deletion	Eap may contribute to pathogenicity by promoting adhesion of whole staphylococcal cells to complex eukaryotic substrates
Palankar et al. (2018) Int. J. Med. Microbiol. [79]	In vitro Single center (Germany)	*S. aureus* Mu50	To investigate Eap subdomain and interaction with platelet	Eap subdomain Eap D3D4 specifically interacts and rapidly activates human platelets
Hussain et al. (2008) Infect. Immun. [80]	In vitro Single center (Germany)	*S*. *aureus* Newman cultures vs. *S*. *aureus* Wood 46	To investigate the interactions of full-length Eap and five recombinant tandem repeat domains with host proteins	More than one Eap tandem repeat domain is required for *S. aureus* agglutination, adherence, and cellular invasion but not for the stimulation of PBMC proliferation
Heying et al. (2007) Thromb. Haemost. [114]	Human Single Center (Germany)	*S. aureus L. lactis* culture cultured human EC	To investigate the role of FnBPA, FnBPB ClfA to promote bacterial adherence to cultured human ECs	*S. aureus* FnBPs, but not ClfA, lead pathogenicity to non-pathogenic *L. lactis*. Adhesins (ICAM-1 and VCAM-1) evokes inflammation (interleukin-8) as well as procoagulant activity
Piroth et al. (2008) Infect. Immun. [115]	Animal model Single Center (Switzerland)	*S. aureus L. lactis* culture In vitro and in vivo	To study the subdomain of FnBPA responsible for fibrinogen and fibronectin binding, cell invasion, and in vivo endocarditis	Fb binding combined with fibronectin binding to synergize the invasion of cultured cell lines is correlated with IE severity
Pappelbaum et al. (2013) Circulation [117]	Human/Animal Single center (Germany)	6 WT mice with VWF vs. 5 knockout mice vs. Cultured human EC	Whether ULVWF mediates bacterial adherence	ULVWF contributes to the initial pathogenic step of *S aureus*-induced endocarditis in patients with an intact endothelium. Heparin or ADAMTS13 intervenes in decreasing ULVWF adherence
Claes et al. (2018) Thromb. Haemost. [119]	Human/Animal Multicenter (Belgium pilot)	*L. lactis-clfA* vs. *L. lactis-fnbpA* vs. Cultured human EC	To study the influence of shear flow and plasma on the binding of ClfA and FnbpA	Pharmacological inhibition of ClfA-Fg interactions may constitute a valuable additive treatment in infective endocarditis
Ko et al. (2016) mBio [120]	Animal model Single Center (USA)	Rat model of IE induced	To identify variants of a linear Fg binding motif, present in Coa and Efb which are responsible for the Fg binding activities of these proteins	*S. aureus* coagulase can induce the formation of a fibrinogen shield in experimental abscess models which surrounds and protects bacteria in the microcolony from clearance
Niemann et al. (2021) mBio [125]	Animal Multicenter (Germany)	Rat model of IE induced in osteoblasts vs. epithelial cells	To demonstrate that *S. aureus* was less engulfed in osteoblasts than in epithelial cells	Large differences of *S. aureus* uptake efficacy in different host cell types. In vivo differences between courses of bacterial infections and the localization of bacteria in different clinical settings mediated by α5β1-integrin
Pietrocola et al. (2020) J. Biol. Chem. [126]	Animal Multicenter center (Italy pilot)	Rat model of IE induced	To evaluate a variety of virulence factors that promote infection by *S. aureus*	Adherence to and invasion of epithelial and ECs by IsdB-expressing *S. aureus* cells was promoted by Vn, and an α_v_β_3_ integrin-blocking mAb
Alfeo et al. (2021) Sci. Rep. [127]	Animal Multicenter center (Italy pilot)	Rat model of IE induced	To study IsdB protein and Vn binding Interacts with vWF	Importance of IsdB in adherence of *S. aureus* to the endothelium colonization and as potential therapeutic target
Ditkowski et al. (2021) J. Thorac. Cardiovasc. Surg. [129]	Human Multicenter (Belgium pilot)	5 graft tissues	To investigate contributions by platelets and plasma fibrinogen to IE initiation on various grafts used for valve replacement	Binding of plasma Fg to especially BJV grafts enables adhesion of single platelets via α_IIb_β_3_. *S aureus* attaches from blood to activated bound platelet α_IIb_β_3_ via plasma fibrinogen

Abbreviations: ADAMTS13, a disintegrin and metalloproteinase with thrombospondin motifs 13; BJV, bovine jugular vein; ClfA, clumping factor A; Eap, *S. aureus* extracellular adhesion protein; EC, endothelial cell; Fc, fibrinogen; FnBPA, fibronectin-binding protein A; IsdB, iron-regulated surface determinant B protein; IL, interleukine; *L. lactis*, *Lactococcus lactis*; SdrE; mAb, monoclonal antibody; PBMC, peripheral blood mononuclear cells; TNF, tumor necrosis factor; ULVWF, ultra-large von Willebrand factor; Vn, extracellular matrix protein vitronectin; vWbp, von Willebrand factor-binding protein; vhp, vWbp homologous protein.

## 4. Conclusions

Several factors allow *Staphylococcus aureus* infections to proliferate within the host with numerous promoting and perpetuating agents with direct bacterial pathogenic activity predominating other factors, such as rheumatic heart disease. This is further supported by the roles of teichoic and lipoteichoic acids within teichoic acid, which favour host cell invasion. The complex interaction with the host’s innate immunity also potentiates its virulence. The role of vaccines has not been successfully translated to the clinical setting thus far. Ameliorating these molecular pathways may soon serve as a therapeutic avenue for the prevention and treatment of these infections, with antiplatelet agents showing promising results.

## Data Availability

Not applicable.

## Supplementary Materials

The following supporting information can be downloaded at: https://www.mdpi.com/article/10.3390/ijms241311068/s1, reference [136] is cited in Supplementary Materials.
